# Association between pre-existing respiratory disease and its treatment, and severe COVID-19: a population cohort study

**DOI:** 10.1016/S2213-2600(21)00095-3

**Published:** 2021-08

**Authors:** Paul Aveyard, Min Gao, Nicola Lindson, Jamie Hartmann-Boyce, Peter Watkinson, Duncan Young, Carol A C Coupland, Pui San Tan, Ashley K Clift, David Harrison, Doug W Gould, Ian D Pavord, Julia Hippisley-Cox

**Affiliations:** a Nuffield Department of Primary Care Health Sciences, University of Oxford, Oxford, UK; b Nuffield Department of Clinical Neurosciences, John Radcliffe Hospital, University of Oxford, Oxford, UK; c Nuffield Department of Medicine, University of Oxford, Oxford, UK; d NIHR Oxford Biomedical Research Centre, Oxford University Hospitals, NHS Foundation Trust, Oxford, UK; e School of Public Health, Peking University Health Science Centre, Beijing, China; f University of Nottingham, Division of Primary Care, Faculty of Medicine & Health Sciences, University Park, Nottingham, UK; g Intensive Care National Audit & Research Centre, London, UK

## Abstract

**Background:**

Previous studies suggested that the prevalence of chronic respiratory disease in patients hospitalised with COVID-19 was lower than its prevalence in the general population. The aim of this study was to assess whether chronic lung disease or use of inhaled corticosteroids (ICS) affects the risk of contracting severe COVID-19.

**Methods:**

In this population cohort study, records from 1205 general practices in England that contribute to the QResearch database were linked to Public Health England's database of SARS-CoV-2 testing and English hospital admissions, intensive care unit (ICU) admissions, and deaths for COVID-19. All patients aged 20 years and older who were registered with one of the 1205 general practices on Jan 24, 2020, were included in this study. With Cox regression, we examined the risks of COVID-19-related hospitalisation, admission to ICU, and death in relation to respiratory disease and use of ICS, adjusting for demographic and socioeconomic status and comorbidities associated with severe COVID-19.

**Findings:**

Between Jan 24 and April 30, 2020, 8 256 161 people were included in the cohort and observed, of whom 14 479 (0·2%) were admitted to hospital with COVID-19, 1542 (<0·1%) were admitted to ICU, and 5956 (0·1%) died. People with some respiratory diseases were at an increased risk of hospitalisation (chronic obstructive pulmonary disease [COPD] hazard ratio [HR] 1·54 [95% CI 1·45–1·63], asthma 1·18 [1·13–1·24], severe asthma 1·29 [1·22–1·37; people on three or more current asthma medications], bronchiectasis 1·34 [1·20–1·50], sarcoidosis 1·36 [1·10–1·68], extrinsic allergic alveolitis 1·35 [0·82–2·21], idiopathic pulmonary fibrosis 1·59 [1·30–1·95], other interstitial lung disease 1·66 [1·30–2·12], and lung cancer 2·24 [1·89–2·65]) and death (COPD 1·54 [1·42–1·67], asthma 0·99 [0·91–1·07], severe asthma 1·08 [0·98–1·19], bronchiectasis 1·12 [0·94–1·33], sarcoidosis 1·41 [0·99–1·99), extrinsic allergic alveolitis 1·56 [0·78–3·13], idiopathic pulmonary fibrosis 1·47 [1·12–1·92], other interstitial lung disease 2·05 [1·49–2·81], and lung cancer 1·77 [1·37–2·29]) due to COVID-19 compared with those without these diseases. Admission to ICU was rare, but the HR for people with asthma was 1·08 (0·93–1·25) and severe asthma was 1·30 (1·08–1·58). In a post-hoc analysis, relative risks of severe COVID-19 in people with respiratory disease were similar before and after shielding was introduced on March 23, 2020. In another post-hoc analysis, people with two or more prescriptions for ICS in the 150 days before study start were at a slightly higher risk of severe COVID-19 compared with all other individuals (ie, no or one ICS prescription): HR 1·13 (1·03–1·23) for hospitalisation, 1·63 (1·18–2·24) for ICU admission, and 1·15 (1·01–1·31) for death.

**Interpretation:**

The risk of severe COVID-19 in people with asthma is relatively small. People with COPD and interstitial lung disease appear to have a modestly increased risk of severe disease, but their risk of death from COVID-19 at the height of the epidemic was mostly far lower than the ordinary risk of death from any cause. Use of inhaled steroids might be associated with a modestly increased risk of severe COVID-19.

**Funding:**

National Institute for Health Research Oxford Biomedical Research Centre and the Wellcome Trust.

## Introduction

During the COVID-19 pandemic, attention has concentrated on risks from cardiometabolic disease; less attention has been focused on chronic respiratory disease, although pre-existing respiratory disease might be expected to worsen COVID-19 disease severity. A review of case series showed that the prevalence of chronic respiratory disease in patients hospitalised with COVID-19 was lower than the prevalence in the general population.^1^ A subsequent systematic review found mixed evidence on asthma, with some data suggesting hospitalisation was equally probable but that COVID-19 would probably be more severe;^2^ however, the evidence was weak. A systematic review and meta-analysis of cohorts of people hospitalised with COVID-19 suggested that chronic obstructive pulmonary disease (COPD) was associated with a four-times higher risk of severe disease (meaning intensive care unit [ICU] admission, death, or other critical outcomes).^3^ However, although useful for prognostication in hospital, such cohorts cannot assess true risk because most people with COVID-19 are not admitted to hospital and decisions about who is and who is not admitted to hospital or to ICU are in part determined by severity of comorbid conditions. Because only a small fraction of people with COVID-19 are admitted to hospitals, selecting only hospitalised patients for cohort entry often induces bias, producing incorrect estimates of the size and even direction of effect of risk factors for deterioration or death.^4^

**Research in context**

**Evidence before this study**

We searched MEDLINE from inception of the database to Nov 21, 2020, using the terms “respiratory disease”, “COVID-19”, and “systematic review”. One review estimated that 5% of all people admitted to hospital with COVID-19 had chronic respiratory disease and a second that 8% of people who progressed to ICU care or who died of COVID-19 had respiratory disease. Two more reviews reported that the odds ratio for death from COVID-19 in people hospitalised with the disease was between two and four. One review focusing only on asthma produced an uncertain estimate. One community cohort reported little or no increased risk of severe COVID-19 in people with asthma, but reported a 63% increased risk of COVID-19 death associated with all other respiratory diseases combined. We also searched MEDLINE from inception of the database to Nov 21, 2020, using the terms “COVID-19” and “inhaled corticosteroids” (ICS). Two systematic reviews have examined the evidence on whether ICS are associated with COVID-19 outcomes. One review found no relevant studies. A second reported that in people admitted to hospital with severe COVID-19, use of ICS was associated with faster recovery and shorter duration of hospital stay, but it found no evidence of association with need for ICU care or death. A community cohort study reported evidence of lower risk of hospital admission with COVID-19 for users of ICS than for people with asthma who do not use ICS. A second community cohort suggested that steroids were associated with a modestly increased risk of death from COVID-19, but residual confounding could have explained the association.

**Added value of this study**

This study used a representative and unselected cohort to generate risk estimates for severe COVID-19 in people with chronic respiratory diseases. In doing so, collider bias could be avoided, which often creates distorted risk estimates when the population studied includes only people with moderately severe COVID-19—defined as admission to hospital. Unlike previous studies of people hospitalised with COVID-19, which suggested large increases in risk, this cohort provides evidence that the risk of severe COVID-19 in people with common airways diseases is only modestly raised over the risk in people without such diseases. Interstitial lung disease appears to increase the risk of severe COVID-19 by around 50%. These relative risks were similar before and after the shielding policy was introduced. Shielding supported everyone with cystic fibrosis, interstitial lung disease, and people with particularly severe chronic obstructive pulmonary disease (COPD) and asthma to remain at home and avoid all social contact. The study provides evidence that the use of ICS is not associated with a substantially increased the risk of severe COVID-19, but nor does it appear to be associated with reduced risk.

**Implications of all the available evidence**

Asthma appears not to be associated with a major increased risk of the severity of COVID-19, whereas COPD and interstitial lung disease are associated with a 50% increased risk of severe COVID-19. The apparent relative risks of severe COVID-19 arising from chronic respiratory disease are less than those of being male and of having diabetes, and are a small fraction of the ordinary risk of death from any cause. Advice about COVID-19 to people with chronic respiratory disease might have inadvertently generated undue anxiety. Placing these modestly increased risks in the context of other COVID-19 risk factors and the normal risk of mortality might reduce anxiety reported by individuals with respiratory diseases. Whether using inhaled steroids is associated with an increased risk of severe COVID-19 remains unclear.

Data are scarce on the associations between other lung diseases and COVID-19. The International Severe Acute Respiratory and Emerging Infection Consortium (ISARIC) examined the risk of death in people hospitalised with interstitial lung diseases. After propensity score matching, the hazard ratio (HR) was 1·60 (95% CI 1·17–2·18).^5^ People with a greater restriction of vital capacity had a somewhat higher mortality. There were few data on the different types of interstitial disease.

Inhaled corticosteroids (ICS) are commonly used treatments for airways disease and they might modify the severity of COVID-19. Studies show reduced coronavirus replication and cytokine production in cell lines treated with steroids and β-agonists.^6^, ^7^ By contrast, there is observational evidence and evidence from randomised trials that ICS are associated with an increased risk of non-COVID-19 respiratory tract infections.^8^, ^9^ A systematic review aimed to assess the association between previous treatment with ICS and COVID-19 severity;^8^ however, no studies were identified. Since then, the OpenSAFELY cohort^10^ reported some evidence of increased risk in patients with COPD and patients with asthma prescribed high-dose ICS, but these differences were plausibly explained by risk factors not recorded in the available data.^10^

Given the dearth of data on true risks of respiratory disease for severe COVID-19, we undertook a cohort study of a large, unselected community-based population to examine these issues.

## Method

### Study design and participants

All adults registered with 1205 general practices in England contributing to the QResearch database (version 44, uploaded March 23, 2020) were included in this population cohort study. The protocol was published and described data and analyses on smoking that will be presented elsewhere.^11^ The data included all general practitioner consultations; illnesses, including those diagnosed in hospital that the general practitioner recorded; and prescriptions in primary care. These were linked to SARS-CoV-2 RT-PCR test records held by Public Health England. The QResearch records were linked to Hospital Episode Statistics, which gave the diagnoses of people admitted to hospitals in England, and the Intensive Care National Audit and Research Centre (ICNARC) Case Mix Programme database. This provided data from all ICUs in England. We also linked the QResearch database to death certificates provided by the Office for National Statistics.

All patients 20 years or older who were registered with one of the 1205 general practices on Jan 24, 2020, were included in the cohort. Patients were censored following the date of death, occurrence of the relevant outcome of interest (either hospital admission, ICU admission, or death), or study end date (April 30, 2020), whichever occurred first. This population cohort study was approved by the QResearch Science Committee, which has delegated power from the NHS ethics committee to approve studies using QResearch.

### Procedures

We examined the risk of severe COVID-19 in relation to COPD, asthma, bronchiectasis, cystic fibrosis, sarcoidosis, extrinsic allergic alveolitis (also known as hypersensitivity pneumonitis), idiopathic pulmonary fibrosis, other interstitial lung disease, and lung cancer compared with people without these diseases. In a post-hoc analysis, risk of severe COVID-19 was assessed in patients with active asthma (defined as having at least one prescription for asthma medication) and severe asthma (defined as being prescribed at least three different classes of medication for asthma in the year before cohort entry). These exposures were derived from the primary care record only and recorded any time since registration in the practice. General practitioners will typically diagnose COPD and asthma, and will probably record all serious diagnoses made in secondary or tertiary care, such as interstitial lung disease. The observed prevalence matched that expected from epidemiological surveys.^12^ We coded a person as exposed to ICS if they were prescribed ICS at least twice within 150 days of the study start date.

We assessed the potential confounders by creating directed acyclic graphs. For the associations between respiratory disease and COVID-19 severity, we identified three causal paths: a direct path and two through treatments, which for these analyses we classified as ICS and other treatments for airways disease (appendix p 1). The potential confounding paths were blocked by controlling for smoking, body-mass index (BMI), and demographic factors (age as a continuous variable, sex, ethnic group, socioeconomic status, and region of England). Because adjusting for smoking status and demographic factors might not completely block confounding, we adjusted for smoking-related and non-smoking-related morbidity, including all other respiratory diseases.

We hypothesised only one causal path between ICS use and severe COVID-19 in the directed acyclic graphs (appendix p 1): respiratory disease. Thus, controlling for this respiratory disease will block all other paths to the outcome. However, as factors related to severity of disease were not recorded, we also adjusted for demographic factors, smoking, BMI, other comorbid conditions, and number of airways medications (an indirect marker). Number of treatments was defined as the number of classes of respiratory drugs prescribed (short-acting β-agonists, long-acting β-agonists, long-acting muscarinic antagonist, leukotriene receptor antagonists, and theophyllines); use of respiratory drugs was determined by two or more prescriptions received within 150 days before cohort entry and classified as three or more versus one or two. All covariates were obtained from general practice records before cohort entry.

### Outcomes

The outcomes were hospital admission, ICU admission, and death due to COVID-19. Hospital admission was defined as a positive test for SARS-CoV-2 and appearing in the Hospital Episode Statistics dataset as an in-patient within 30 days of that test or having an International Classification of Diseases (ICD)-10 code U07.1 for confirmed COVID-19 or U07.2 for suspected COVID-19. Admission to ICU was defined as admission to an ICU with severe COVID-19 (ICD-10 code U07.1 or U07.2) in ICNARC records. Death from COVID-19 was defined as having confirmed or suspected COVID-19 (ICD-10 codes U07.1 and U07.2) on the death certificate, including deaths in and out of hospital.

Residents of care homes are almost all extremely frail, which is the strongest risk factor for adverse outcomes from SARS-CoV-2 infection.^13^ Although respiratory disease might cause frailty and the need for care, the experience of this group is not representative of most people with underlying respiratory disease. In a post-hoc analysis, we analysed death from COVID-19 excluding all care home residents.

We examined the effect of the national lockdown and shielding introduced in England on March 23, 2020. Lockdown was a legal requirement for everyone except essential workers to stay at home, with dispensation for essential shopping and exercising. Shielding asked people with diseases or medications that were assumed to convey extreme risk of severe COVID-19 to stay at home at all times, with support provided to achieve this. Shielding also required practisers to minimise any social contact, even with members of the same household.

We assessed whether there was evidence that the associations between two common respiratory diseases, COPD and asthma, and severe COVID-19 were modified by age, sex, ethnicity, and smoking status.

We calculated representative absolute death rates from COVID-19 for people of various ages with and without respiratory disease, presenting the difference. To put the mortality rates into context, we compared excess deaths in people with respiratory diseases with so-called normal risk.

### Statistical analysis

We used Cox's proportional hazards models to estimate the mutually adjusted HRs for the associations between each respiratory disease and each of the severe COVID-19 outcomes and to estimate the mutually adjusted HRs between ICS use and severe COVID-19, with Stata (version 16.0). HRs were adjusted for patient age, sex, demographic factors, and comorbidities in all analyses. When adjustment variables had missing values, we included these participants in a missing category.

In the post-hoc analyses, we excluded people living in care homes and reran the analysis to examine the risk of death from COVID-19. To assess the effects of shielding, we reassessed hospitalisation, ICU admissions, and deaths, censoring outcomes on March 31, 2020, for the preshielding period, and from April 1–30, 2020, for the post-shielding period. To assess the effect of age, sex, ethnicity, and smoking status on the association between COPD and asthma and COVID-19, we added appropriate multiplicative interaction terms to the fully adjusted model described.

In the assessment of absolute death rates, Spiegelhalter defined normal risk as the risk of death from any cause that a person would face in the same time period,^14^ which was calculated with data from the Office for National Statistics.^15^ For asthma and COPD, we present the risks assuming no interaction with age and then assuming age-specific risks.

### Role of the funding source

The study was funded by the National Institute for Health Research Oxford Biomedical Research Centre and the Wellcome Trust. The funders had no role in study design; data collection, analysis, and interpretation; writing of the Article; and the decision to submit it for publication.

## Results

Between Jan 24 and April 30, 2020, 8 256 161 adults were included in the cohort. The most common respiratory diseases were asthma (1 090 028 [13·2%] individuals), COPD (193 520 [2·3%] individuals), and bronchiectasis (41 271 [0·5%]; appendix p 2; table 1). During follow up, 14 479 (0·2%) of 8 256 161 adults were admitted to hospital with COVID-19, 1542 (<0·1%) were admitted to ICU, and 5956 (0·1%) died of COVID-19. In the whole population, 1 271 108 (15·4%) had any respiratory disease. People with underlying respiratory disease gave rise to 3696 (25·5%) of 14 479 hospital admissions, 275 (17·8%) of 1542 ICU admissions, and 1485 (24·9%) of 5956 deaths (appendix pp 2–7; Table 1, Table 2).

**Table 1:** Risk of severe COVID-19 outcomes for people with underlying respiratory disease

	**Number of patients with outcome (n [%]/N)**	Unadjusted HR (95% CI)*	**HR (95% CI) adjusted for age and sex**	**HR (95% CI) also adjusted for other demographic factors**†	**HR (95% CI) also adjusted for comorbidities**‡
**Hospitalisation**					
COPD	1555 (0·8%)/193 520	5·09 (4·83–5·36)	1·85 (1·75–1·95)	1·79 (1·70–1·90)	1·54 (1·45–1·63)
Asthma	2266 (0·2%)/1 090 028	1·22 (1·17–1·28)	1·39 (1·33–1·46)	1·32 (1·26–1·38)	1·18 (1·13–1·24)
Active asthma	1720 (0·3%)/535 126	1·95 (1·85– 2·05)	1·56 (1·48–1·64)	1·43 (1·35–1·50)	1·26 (1·20–1·33)
Severe asthma	1369 (0·4%)/385 702	2·14 (2·02–2·26)	1·65 (1·56–1·75)	1·47 (1·39–1·55)	1·29 (1·22–1·37)
Bronchiectasis	319 (0·8%)/41 271	4·53 (4·06–5·07)	1·70 (1·52–1·90)	1·67 (1·49–1·87)	1·34 (1·20–1·50)
Cystic fibrosis	5 (0·2%)/2081	1·37 (0·57–3·30)	1·62 (0·67–3·89)	1·78 (0·74–4·28)	1·55 (0·65–3·73)
Sarcoidosis	84 (0·5%)/17 624	2·74 (2·21–3·39)	1·74 (1·40–2·15)	1·53 (1·23–1·90)	1·36 (1·10–1·68)
Extrinsic allergic alveolitis	16 (0·7%)/2331	3·97 (2·43–6·48)	1·75 (1·07–2·85)	1·86 (1·14–3·03)	1·35 (0·82–2·21)
Idiopathic pulmonary fibrosis	110 (1·5%)/7454	8·80 (7·29–10·62)	2·40 (1·99–2·89)	2·28 (1·89–2·75)	1·59 (1·30–1·95)
Other interstitial lung diseases	73 (1·3%)/5677	7·57 (6·02–9·53)	2·54 (2·02–3·20)	2·43 (1·93–3·05)	1·66 (1·30–2·12)
Lung cancer	139 (1·3%)/10 792	7·92 (6·70–9·36)	2·73 (2·31–3·23)	2·63 (2·22–3·11)	2·24 (1·89–2·65)
**ICU admission**§					
COPD	59 (<0·1%)/193 520	1·68 (1·29–2·18)	0·85 (0·65–1·11)	0·92 (0·70–1·20)	0·89 (0·68–1·17)
Asthma	213 (<0·1%)/1 090 028	1·05 (0·91–1·22)	1·18 (1·02–1·36)	1·09 (0·95–1·27)	1·08 (0·93–1·25)
Active asthma	165 (<0·1%)/535 126	1·73 (1·47–2·03)	1·62 (1·37–1·90)	1·36 (1·16–1·61)	1·34 (1·14–1·58)
Severe asthma	124 (<0·1%)/385 702	1·79 (1·49–2·15)	1·64 (1·37–1·98)	1·33 (1·10–1·60)	1·30 (1·08–1·58)
Bronchiectasis	18 (<0·1%)/41 271	2·37 (1·49–3·78)	1·36 (0·85–2·17)	1·46 (0·91–2·33)	1·47 (0·91–2·36)
Sarcoidosis	10 (0·1%)/17 624	3·06 (1·64–5·70)	2·22 (1·19–4·14)	1·65 (0·89–3·08)	1·51 (0·81–2·81)
Idiopathic pulmonary fibrosis	6 (0·1%)/7454	4·48 (2·01–9·99)	1·87 (0·84–4·18)	1·88 (0·84–4·19)	1·97 (0·85–4·55)
**Death**¶					
COPD	811 (0·4%)/193 520	6·66 (6·19–7·18)	1·82 (1·69–1·96)	1·64 (1·51–1·77)	1·54 (1·42–1·67)
Asthma	762 (0·1%)/1 090 028	0·96 (0·89–1·04)	1·19 (1·1–1·28)	1·12 (1·04–1·21)	0·99 (0·91–1·07)
Active asthma	602 (0·1%)/535 126	1·62 (1·49–1·77)	1·28 (1·18–1·39)	1·18 (1·09–1·29)	1·05 (0·96–1·15)
Severe asthma	476 (0·1%)/385 702	1·78 (1·62–1·95)	1·35 (1·23–1·48)	1·21 (1·11–1·34)	1·08 (0·98–1·19)
Bronchiectasis	138 (0·3%)/41 271	4·77 (4·03–5·65)	1·35 (1·14–1·60)	1·29 (1·09–1·52)	1·12 (0·94–1·33)
Sarcoidosis	32 (0·2%)/17 624	2·53 (1·79–3·58)	1·63 (1·15–2·31)	1·58 (1·11–2·23)	1·41 (0·99–1·99)
Extrinsic allergic alveolitis	8 (0·3%)/2331	4·82 (2·41–9·65)	1·75 (0·87–3·50)	2·02 (1·01–4·03)	1·56 (0·78–3·13)
Idiopathic pulmonary fibrosis	62 (0·8%)/7454	12·09 (9·42–15·53)	2·14 (1·66–2·74)	2·04 (1·58–2·62)	1·47 (1·12–1·92)
Other interstitial lung diseases	45 (0·8%)/5677	11·37 (8·48–15·25)	2·7 (2·01–3·62)	2·71 (2·02–3·63)	2·05 (1·49–2·81)
Lung cancer	60 (0·6%)/10 792	8·33 (6·46–10·74)	2·18 (1·69–2·81)	1·95 (1·51–2·51)	1·77 (1·37–2·29)

Individuals could have more than one respiratory disease. COPD=chronic obstructive pulmonary disease. HR=hazard ratio. ICU=intensive care unit.

* Adjusted for all other respiratory diseases.

† Also adjusted for ethnicity, socioeconomic status, region of England, body-mass index (categorical variable), and smoking status (with current intensity of smoking as categorical variables).

‡ Also adjusted for non-smoking-related illness (hypertension, type 1 diabetes, chronic liver disease, chronic neurological disease) and smoking-related illness (coronary heart disease, stroke, atrial fibrillation, type 2 diabetes, chronic kidney disease).

§ Data for people with cystic fibrosis, sarcoidosis, extrinsic allergic alveolitis, idiopathic pulmonary fibrosis, other interstitial lung disease, and lung cancer not presented because fewer than five people had the outcome.

¶ Data for people with cystic fibrosis not presented because there were no deaths in this group.

**Table 2:** The association between respiratory disease and risk of COVID-19 death excluding care home residents†

	**Number of patients who died (n [%]/N)**	HR (95% CI)*
COPD	627 (0·3%)/189 533	1·55 (1·41–1·70)
Asthma	617 (0·1%)/1 084 522	1·05 (0·96–1·15)
Active asthma	502 (0·1%)/531 517	1·13 (1·03–1·25)
Severe asthma	400 (0·1%)/382 987	1·15 (1·04–1·28)
Bronchiectasis	112 (0·3%)/40 633	1·20 (0·99–1·45)
Sarcoidosis	25 (0·1%)/17 507	1·37 (0·92–2·03)
Extrinsic allergic alveolitis	6 (0·3%)/2309	1·40 (0·63–3·15)
Idiopathic pulmonary fibrosis	50 (0·7%)/7252	1·54 (1·14–2·08)
Other interstitial lung diseases	41 (0·7%)/5589	2·37 (1·70–3·31)
Lung cancer	53 (0·5%)/10 535	2·07 (1·57–2·72)

People with active asthma had at least one prescription for asthma medication. People with severe asthma were prescribed at least three different classes of medication for asthma in the year before cohort entry. COPD=chronic obstructive pulmonary disease. HR=hazard ratio.

* Adjusted for demographic factors (age, gender, ethnicity, socioeconomic status, region of England), body-mass index (categorical variable), smoking status, non-smoking-related illness (hypertension, type 1 diabetes, chronic liver disease, and chronic neurological disease), smoking-related illness (coronary heart disease, stroke, atrial fibrillation, type 2 diabetes, and chronic kidney disease), and all other respiratory diseases.

† Data for people with cystic fibrosis not presented as nobody with cystic fibrosis died.

Adjusted for age and sex only, all respiratory diseases were associated with increased risk of hospitalisation with COVID-19 compared with those without respiratory diseases (table 1; appendix p 2). People with asthma had the lowest risk, with a 39% increase in risk compared with people without asthma, whereas people with lung cancer were more than twice as likely to be hospitalised with COVID-19. After adjustment for other demographic factors, BMI, smoking status, and other comorbidities, these risks decreased; however, people with respiratory diseases were still at increased risk of hospitalisation (HR 1·54 [95% CI 1·45–1·63] for COPD, 1·18 [1·13–1·24] for asthma, 1·29 [1·22–1·37] for severe asthma, 1·34 [1·20–1·50] for bronchiectasis, 1·36 [1·10–1·68] for sarcoidosis, 1·35 [0·82–2·21] for extrinsic allergic alveolitis, 1·59 [1·30–1·95] for idiopathic pulmonary fibrosis, 1·66 [1·30–2·12] for other interstitial lung disease, and 2·24 [1·89–2·65] for lung cancer). The HRs indicated risk of hospitalisation for most respiratory diseases was increased by between 30% and 50%, with an 18% increase in risk for all people with asthma. In people with active asthma, there was a 26% increased risk, and for severe asthma, a 29% increased risk. People with lung cancer were at twice the risk of admission to hospital compared with people without lung cancer.

People with interstitial lung diseases and cystic fibrosis were very rarely admitted to ICU and so the HRs were not calculated. After full adjustment, there was no evidence that people with asthma, COPD, or bronchiectasis were more likely to be admitted to ICU than the people without these conditions. The HR for asthma was 1·08 (95% CI 0·93–1·25); however, people with active asthma (34%) and severe asthma (30%; HR 1·30 [1·08–1·58]) were at higher risk of ICU admission (table 1).

Having COPD was associated with a 54% (HR 1·54 [95% CI 1·42–1·67]) increase in the risk of death due to COVID-19 in the fully adjusted model, whereas there was no evidence that people with asthma were at an increased risk of death (0·99 [0·91–1·07]), even for people with active asthma (1·05 [0·96–1·15]) and severe asthma (1·08 [0·98–1·19]). Most interstitial lung diseases were associated with a 50% increased risk of death, but the estimates were imprecise (1·41 [0·99–1·99] for those with sarcoidosis, 1·56 [0·78–3·13] for those with extrinsic allergic alveolitis, 1·47 [1·12–1·92] for those with idiopathic pulmonary fibrosis, and 2·05 [1·49–2·81] for those with other interstitial lung disease). The risk of death in people with lung cancer (1·77 [1·37–2·29]) was 77% higher than in the general population (table 1). HR for death in individuals with bronchiectasis was 1·12 [0·94–1·33]. No one with cystic fibrosis died because of COVID-19.

1565 (3·1%) of 50 769 residents in care homes died due to COVID-19, which accounted for 26·3% of all deaths from COVID-19. Therefore, in a post-hoc analysis, we reanalysed the death outcome, excluding individuals living in care homes. Because care home residents gave rise to 1264 (8·7%) of 14479 hospitalisations and 12 (0·8%) of 1542 ICU admissions, we did not reanalyse these outcomes. This sensitivity analysis showed that the HRs for COVID-19-related death associated with respiratory disease were generally lower in the population outside of care homes than in the whole population (table 2).

6857 hospital admissions, 655 ICU admissions, and 905 deaths occurred before shielding guidance could have modified risk for people with underlying respiratory disease. After the implementation of shielding guidance, 7622 hospital admissions, 887 ICU admissions, and 5051 deaths occurred. The association between previous respiratory disease and severe COVID-19 was generally similar before and after shielding was introduced (table 3).

**Table 3:** Modification of the association between respiratory disease and severe COVID-19 by the introduction of lockdown and shielding

	**Overall**	Associations reflecting infection before shielding*	Associations reflecting infection after shielding*
**Hospitalisation**			
COPD	1·54 (1·45–1·63)	1·54 (1·45–1·64)	1·53 (1·45–1·63)
Asthma	1·18 (1·13–1·24)	1·20 (1·14–1·26)	1·17 (1·11–1·28)
Active asthma	1·26 (1·20–1·33)	1·28 (1·21–1·35)	1·25 (1·20–1·33)
Severe asthma	1·29 (1·22–1·37)	1·30 (1·22–1·38)	1·28 (1·20–1·39)
Bronchiectasis	1·34 (1·20–1·50)	1·36 (1·21–1·53)	1·33 (1·19–1·49)
Cystic fibrosis	1·55 (0·65–3·73)	1·65 (0·69–3·97)	1·55 (0·62–3·81)
Sarcoidosis	1·36 (1·10–1·68)	1·37 (1·10–1·70)	1·25 (0·99–1·58)
Extrinsic allergic alveolitis	1·35 (0·82–2·21)	1·36 (0·83–2·21)	1·22 (0·70–2·10)
Idiopathic pulmonary fibrosis	1·59 (1·30–1·95)	1·60 (1·31–1·95)	1·54 (1·24–1·91)
Other interstitial lung diseases	1·66 (1·30–2·12)	1·67 (1·30–2·13)	1·62 (1·25–2·11)
Lung cancer	2·24 (1·89–2·65)	2·31 (1·94–2·75)	2·23 (1·88–2·64)
**ICU admission**†			
COPD	0·89 (0·68–1·17)	0·89 (0·68–1·17)	0·80 (0·59–1·09)
Asthma	1·08 (0·93–1·25)	1·10 (0·94–1·28)	1·07 (0·91–1·27)
Active asthma	1·34 (1·14–1·58)	1·37 (1·14–1·63)	1·32 (1·14–1·59)
Severe asthma	1·30 (1·08–1·58)	1·36 (1·06–1·59)	1·29 (1·08–1·60)
Bronchiectasis	1·47 (0·91–2·36)	1·49 (0·91–2·35)	1·33 (0·78–2·27)
Sarcoidosis	1·51 (0·81–2·81)	1·53 (0·82–2·81)	1·19 (0·57–2·51)
Idiopathic pulmonary fibrosis	1·97 (0·85–4·55)	2·35 (1·01–5·47)	1·96 (0·85–4·54)
**Death**‡			
COPD	1·54 (1·42–1·67)	1·55 (1·31–1·68)	1·49 (1·36–1·63)
Asthma	0·99 (0·91–1·07)	0·98 (0·91–1·07)	1·01 (0·93–1·10)
Active asthma	1·05 (0·96–1·15)	1·04 (0·97–1·15)	1·06 (0·95–1·17)
Severe asthma	1·08 (0·98–1·19)	1·07 (0·98–1·19)	1·09 (0·97–1·21)
Bronchiectasis	1·12 (0·94–1·33)	1·13 (0·98–1·42)	1·11 (0·93–1·34)
Sarcoidosis	1·41 (0·99–1·99)	1·42 (1·00–2·00)	1·22 (0·80–1·86)
Extrinsic allergic alveolitis	1·56 (0·78–3·13)	1·57 (0·78–3·14)	1·42 (0·36–2·56)
Idiopathic pulmonary fibrosis	1·47 (1·12–1·92)	1·48 (1·11–1·92)	1·30 (0·94–1·77)
Other interstitial lung diseases	2·05 (1·49–2·81)	2·06 (1·50–2·82)	2·04 (1·48–2·95)
Lung cancer	1·77 (1·37–2·29)	1·89 (1·43–2·48)	1·76 (1·36–2·27)

Data are hazard ratio (95% CI). People with active asthma had at least one prescription for asthma medication. People with severe asthma were prescribed at least three different classes of medication for asthma in the year before cohort entry. COPD=chronic obstructive pulmonary disease. ICU=intensive care unit.

* Adjusted for demographic factors (age, gender, ethnicity, socioeconomic status, and region of England), body-mass index (categorical variable), smoking status, non-smoking-related illness (hypertension, type 1 diabetes, chronic liver disease, and chronic neurological disease), smoking-related illness (coronary heart disease, stroke, atrial fibrillation, type 2 diabetes, and chronic kidney disease), and all other respiratory diseases.

† Data for people with cystic fibrosis, sarcoidosis, extrinsic allergic alveolitis, idiopathic pulmonary fibrosis, other interstitial lung disease, and lung cancer not presented because fewer than five people had the outcome.

‡ Data for people with cystic fibrosis not presented as there were no deaths in this group.

**Figure 1:**
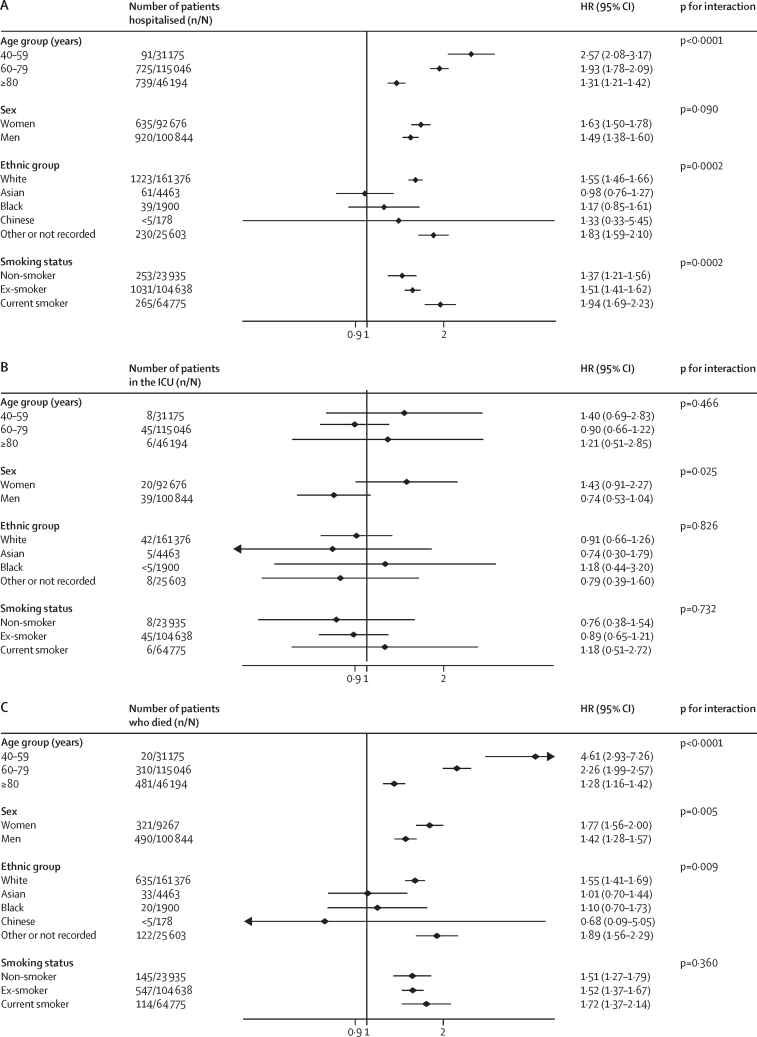
Association between COPD and severe COVID-19 on hospitalisation (A), ICU admission (B), and death (C) COPD=chronic obstructive pulmonary disease. HR=hazard ratio. ICU=intensive care unit.

**Figure 2:**
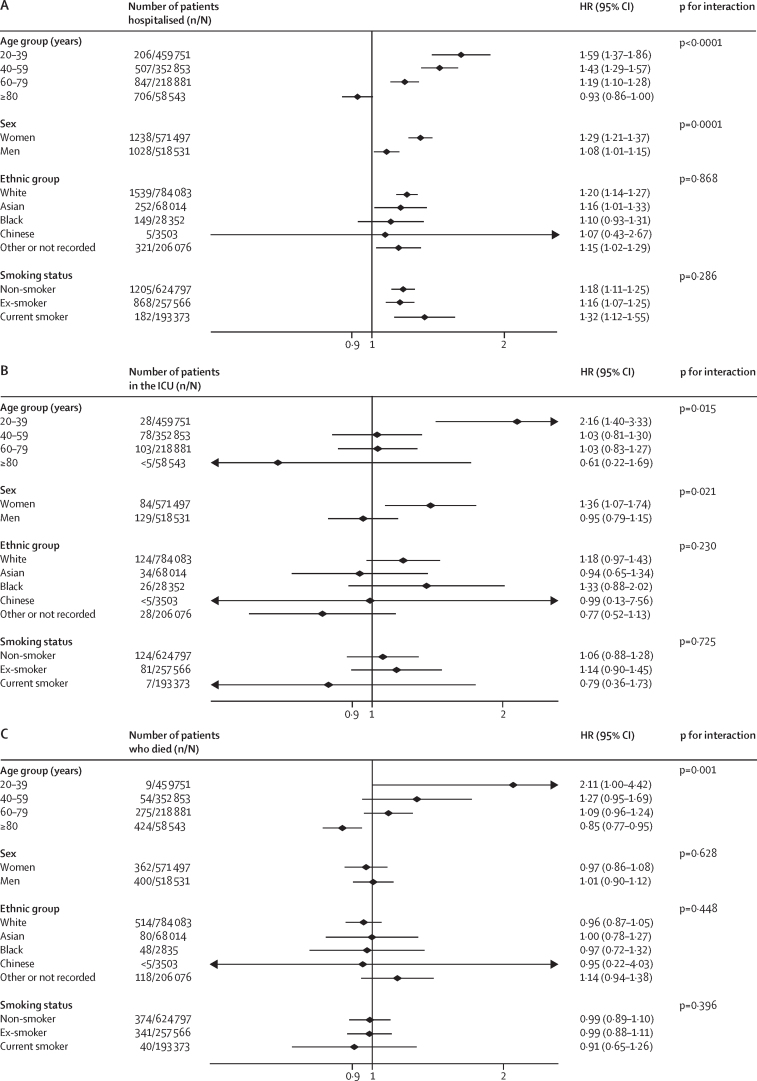
Association between asthma and severe COVID-19 on hospitalisation (A), ICU admission (B), and death (C) HR=hazard ratio. ICU=intensive care unit.

There was also a consistent pattern of effect modification by sex for both asthma and COPD (Figure 1, Figure 2). Women with these diseases were at statistically significantly higher relative hazard of hospitalisation, ICU admission, and death compared with men for all but one analysis.

The risks of hospitalisation, ICU admission, and death due to COVID-19 for people with asthma appeared similar for each ethnic group (figure 2). For COPD, there was evidence that the risk varied by ethnicity. The risks for hospitalisation and death associated with COPD were higher in white individuals than all other ethnic groups, except the group without recorded ethnicity (figure 1). There was no evidence of an effect modification for ICU admission.

**Figure 3:**
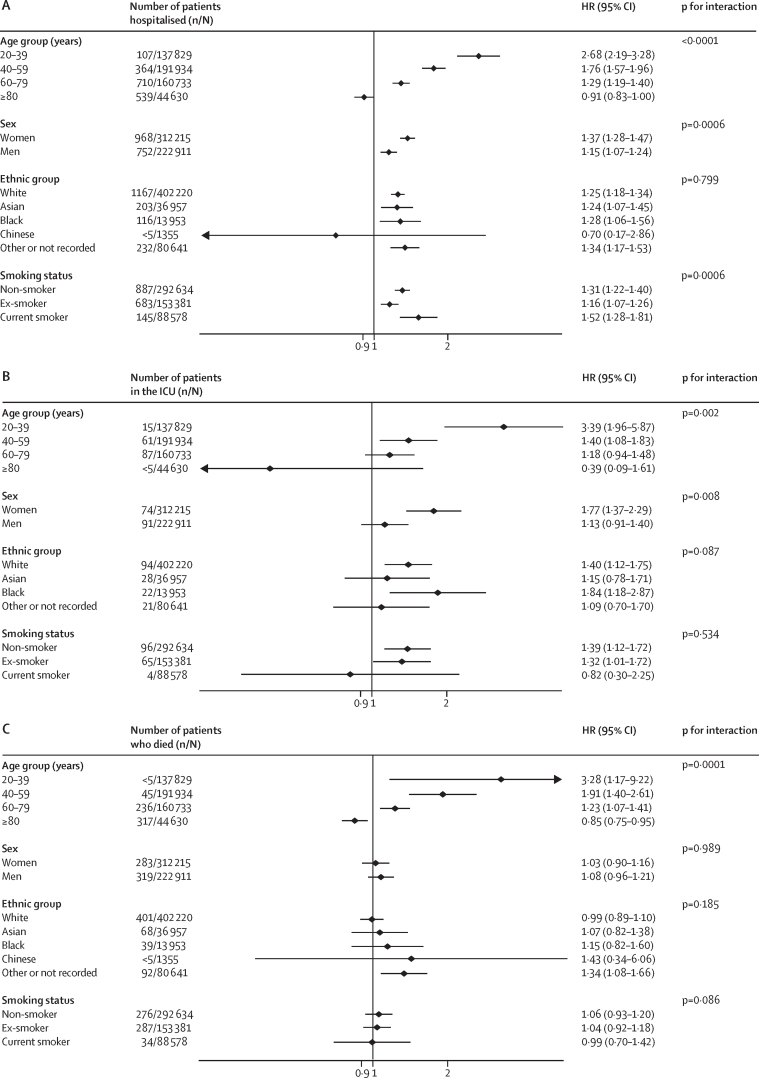
Association between active asthma and severe COVID-19 on hospitalisation (A), ICU admission (B), and death (C) People with active asthma had at least one prescription for asthma medication in the year before cohort entry. HR=hazard ratio. ICU=intensive care unit.

**Figure 4:**
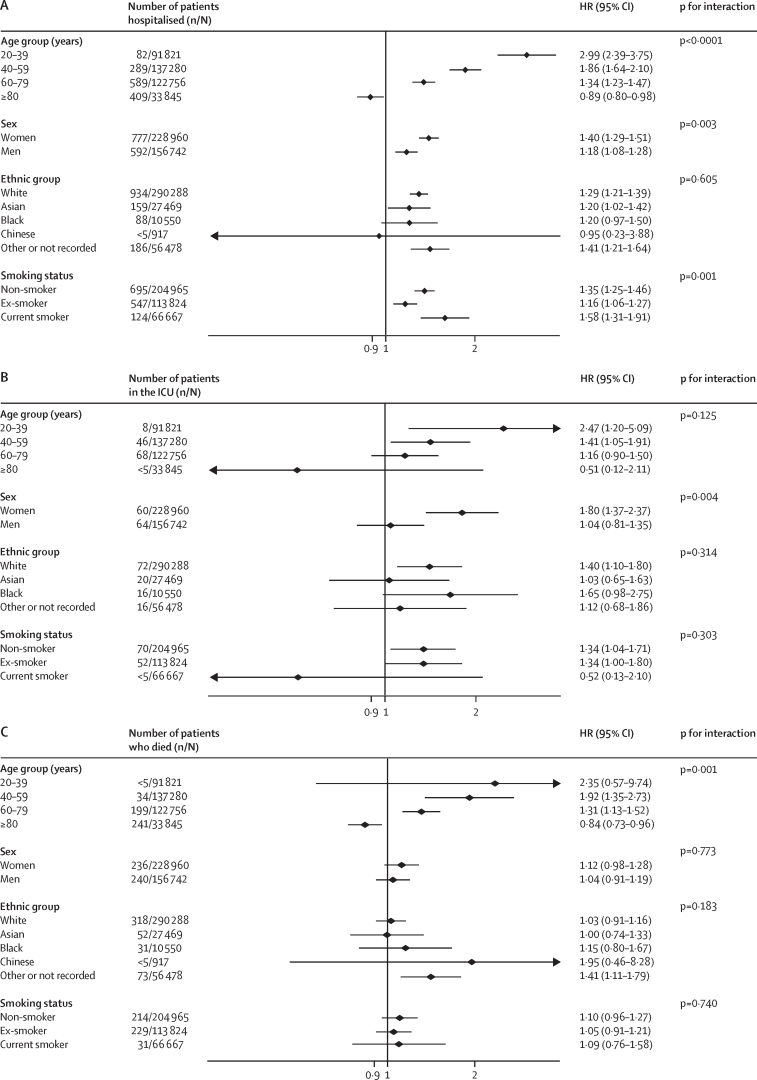
Association between severe asthma and severe COVID-19 on hospitalisation (A), ICU admission (B), and death (C) People with severe asthma were prescribed at least three different classes of medication for asthma in the year before cohort entry. HR=hazard ratio. ICU=intensive care unit.

The HR associated with asthma was significantly modified by age (figure 2). For people younger than 40 years, asthma was associated with a 59% increased risk of hospitalisation, and a more than doubling in the risk of ICU admission and death, but the HRs for asthma in individuals 40 years or older were lower (figure 2). The same pattern was evident in people with COPD (figure 1), active asthma (figure 3), and severe asthma (figure 4). To investigate this pattern, we looked for risk factor differences between people admitted to hospital with asthma or COPD and people who were not that varied by age. In people younger than 40 years, the mean BMI of people admitted to hospital with COVID-19 was 29·5 kg/m^2^, of those admitted to ICU was 33·0 kg/m^2^, and for people who died was 30·9 kg/m^2^, compared with 25·9 kg/m^2^ in people younger than 40 years without severe COVID-19. These BMI differences between people with and without severe COVID-19 got progressively smaller with each age group above that, with no difference in BMI apparent in people older than 80 years with and without severe COVID-19, mirroring the age-specific estimates of risk of severe COVID-19.

There was little evidence that smoking modified the risks associated with asthma and COPD (Figure 1, Figure 2). People who smoked and had COPD were at higher risk of admission to hospital with COVID-19 than people who were not currently smoking but had COPD (figure 1). However, there was no evidence of similar effect modification for ICU admission or death due to COVID-19 (figure 1). There was no evidence that smoking was associated with modified risk in people with asthma (figure 2).

The absolute risk of death from COVID-19 for people with respiratory disease compared with those without respiratory disease was low in absolute terms (table 4). In particular, risk of death was lower than the normal risk of death in all groups, except for people with COPD aged 40–59 years. However, for most other diseases and age groups, the excess risk of death from COVID-19 experienced by people with respiratory disease was far lower than the risk of death between January and April, 2020, for the general population.

**Table 4:** Absolute difference in risk of death from COVID-19 in people with respiratory disease compared with people without respiratory disease and percentage of normal risk

	**Assuming hazard ratio constant with age**		**Assuming hazard ratio differs by age**	
	Mortality from COVID-19 per 100 000 population*	Percentage of normal risk†	Mortality from COVID-19 per 100 000 population*	Percentage of normal risk†
**COPD**				
40–59 years	7	17%	48	117%
60–79 years	56	12%	130	28%
≥80 years	389	38%	202	20%
**Asthma**				
20–39 years	0	0%	1	5%
40–59 years	0	0%	4	10%
60–79 years	0	0%	9	2%
≥80 years	0	−1%	−108‡	−11%‡
**Active asthma**				
20–39 years	0	0%	3	15%
40–59 years	1	2%	12	29%
60–79 years	5	1%	24	5%
≥80 years	36	4%	−115‡	−11%‡
**Severe asthma**				
20–39 years	0	0%	2	10%
40–59 years	1	2%	12	29%
60–79 years	8	2%	32	7%
≥80 years	58	6%	−115‡	−11%‡
**Bronchiectasis**				
20–39 years	0	0%	..	..
40–59 years	2	5%	..	..
60–79 years	12	3%	..	..
≥80 years	86	8%	..	..
**Sarcoidosis**				
20–39 years	1	5%	..	..
40–59 years	5	12%	..	..
60–79 years	42	9%	..	..
≥80 years	295	29%	..	..
**Extrinsic allergic alveolitis**				
20–39 years	1	5%	..	..
40–59 years	7	17%	..	..
60–79 years	58	13%	..	..
≥80 years	403	40%	..	..
**Idiopathic pulmonary fibrosis**				
20–39 years	1	5%	..	..
40–59 years	6	15%	..	..
60–79 years	49	11%	..	..
≥80 years	338	33%	..	..
**Other interstitial lung diseases**				
20–39 years	1	5%	..	..
40–59 years	14	34%	..	..
60–79 years	109	24%	..	..
≥80 years	756	74%	..	..
**Lung cancer**				
20–39 years	1	5%	..	..
40–59 years	10	24%	..	..
60–79 years	80	17%	..	..
≥80 years	555	55%	..	..

People with active asthma had at least one prescription for asthma medication. People with severe asthma were prescribed at least three different classes of medication for asthma in the year before cohort entry. COPD=chronic obstructive pulmonary disease.

* Difference in absolute mortality between people without compared with those with respiratory disease.

† Percentage of normal risk is the COVID-19 deaths divided by absolute risk of death from any cause over 4 months, where all-cause mortality is based on data from 2017–2019; the normal mortality rate for a 4-month period of people aged 20–39 is 19·4 per 100 000, for people aged 40–59 is 40·9 per 100 000, for people aged 60–79 is 460·1 per 100 000, and for people aged 80 years and older is 1015·1 per 100 000.

‡ Numbers are negative because the risk of COVID-19 mortality in people older than 80 years with asthma or COPD is lower than in people without such diseases.

450 784 (5·5%) people in the whole population were using ICS regularly compared with 89 605 (46·3%) of 193 520 people with COPD; 368 496 (33·8%) of 1 090 028 people with asthma; 368 496 (68·9%) of 535 126 people with active asthma; and 316 719 (82·1%) of 385 702 people with severe asthma. Of the 450 784 people using ICS regularly, 342 982 (76·1%) were using at least three airways medications. Overall, there was evidence that use of ICS was associated with a modest risk of severe COVID-19 independently of underlying respiratory disease (HR 1·13 [1·03–1·23] for hospitalisation, 1·63 [1·18–2·24] for ICU admission, and 1·15 [1·01–1·31] for death; table 5); however, these risks were reduced when other risk factors were taken into account.

**Table 5:** Association between regular use of inhaled corticosteroids and severe COVID-19

	**Unadjusted HR (95% CI)**	HR (95% CI) adjusted for presence of respiratory disease*	**HR (95% CI) also adjusted for demographic factors**†	**HR (95% CI) also adjusted for smoking-related morbidity**‡
Hospital admission	2·72 (2·60–2·85)	2·06 (1·94–2·19)	0·97 (0·89–1·05)	1·13 (1·03–1·23)
ICU admission	2·10 (1·78–2·46)	2·52 (2·03–3·13)	1·64 (1·20–2·23)	1·63 (1·18–2·24)
Death	2·63 (2·44–2·84)	2·04 (1·85–2·25)	0·94 (0·83–1·07)	1·15 (1·01–1·31)

People with active asthma had at least one prescription for asthma medication. People with severe asthma were prescribed at least three different classes of medication for asthma in the year before cohort entry. HR=hazard ratio. ICU=intensive care unit.

* Adjusted for presence of chronic obstructive pulmonary disease, asthma, bronchiectasis, sarcoidosis, extrinsic allergic alveolitis, idiopathic pulmonary fibrosis, cystic fibrosis, other interstitial lung disease, and lung cancer.

† Adjusted for sex, age, ethnicity, socioeconomic status, region of England, body-mass index, and smoking status as categorical variables, and number of airways medications (3 or more or fewer).

‡ Adjusted for non-smoking-related illness (hypertension, type 1 diabetes, chronic liver disease, and chronic neurological disease) and smoking-related illness (coronary heart disease, stroke, atrial fibrillation, type 2 diabetes, and chronic kidney disease).

## Discussion

People with pre-existing respiratory disease had a modestly increased risk of severe COVID-19 in this representative community cohort. People with asthma had an 18% increased risk of hospital admission with COVID-19, but there was no evidence that asthma was associated with an increased risk of death. Even in people with asthma who were prescribed three or more airways medications, the risk of death was modestly raised at worst compared with people without respiratory conditions. In the whole population, COPD was associated with a 54% increase in the risk of hospitalisation or death. The risks in people with bronchiectasis were modestly above one, although they were imprecisely estimated for death. Cystic fibrosis was too uncommon to estimate risks with precision, with only five of the 2081 patients in our cohort having a severe outcome, and no one with cystic fibrosis died from COVID-19. Interstitial lung disease was associated with an increased risk of severe COVID-19, while lung cancer was associated with a near doubling of severe COVID-19. People using ICS were at modestly increased risk of severe COVID-19 adjusted for respiratory disease and other comorbidities.

In this large representative sample of the general population, there was previous recording of exposures and potential confounders. As such, this study is likely to have more complete capture of exposures and confounders than studies of patients who were hospitalised, which might be affected by patients' poor recall or the urgency of caring for people with severe COVID-19. Moreover, hospital-based cohorts can distort the strength of association between an exposure and severe COVID-19 through collider bias,^16^ a factor that did not apply here. However, our exposures were restricted to the presence or absence of respiratory disease and did not record its severity. ISARIC reported that interstitial lung disease with greater restrictive lung function was associated with worse outcomes.^5^ Our estimates of association are likely to overestimate the risks in people with mild disease and underestimate them in people with more severe disease. General practice records capture all prescriptions issued by general practitioners. Our classification of people as regular ICS users if they received at least two prescriptions in the 150 days before cohort entry might have classified some regular users as non-users, and some people fulfilling that definition might not have been using steroids at the relevant time of exposure. Exposure misassignment is unlikely to relate to an acute outcome like severe COVID-19 and thus is likely to move estimated associations towards the null. Additionally, our use of ICU care to indicate severe disease, in common with many studies, is a limitation. Most people who worsen with COVID-19 will not be admitted to ICU because their underlying health state suggests little likelihood of benefit. As such, low rates of ICU admission for people with COPD, interstitial lung disease, and lung cancer probably reflect the selection process and not the severity of COVID-19. A final limitation is our definition of severe COVID-19. So-called long COVID is an important outcome of COVID-19, but our dataset is unable to capture risk of this condition.^17^ People with asthma might be at increased risk of longer duration of symptoms from COVID-19.^18^

Because of an expectation of increased risk, it is possible that people with underlying respiratory disease could have more rigorously instituted behavioural measures to reduce their risk of infection from SARS-CoV-2 than people without respiratory conditions. This might mean we have underestimated the true risk of severe COVID-19. The introduction of shielding meant that people with interstitial lung disease and cystic fibrosis probably had less social contact than people without these diseases, and the risks associated with these conditions might have been expected to fall, but we saw only a small amount of evidence supporting this. Our estimates of relative risk for people with underlying respiratory disease reflect a combination of the risk conferred by that disease and the social distancing that people with respiratory disease might have instituted, which might have been more vigorous than that of the general population with or without shielding.

There are few data on the association between respiratory disease and COVID-19. A systematic review reported on 21 studies (n=12 976), all of which comprised cohorts of patients defined by admission to hospital with outcomes representing worsening illness, need for ICU care, and death.^3^ The odds ratio (OR) for severe COVID-19 with any form of respiratory disease was 4·2 (95% CI 2·9–6·0), with no differentiation between respiratory diseases. Selecting only people with COVID-19 that is severe enough to warrant hospital admission means collider bias could have distorted these associations. The prevalence of people with respiratory disease in patients hospitalised with COVID-19 appears to be lower than the prevalence in the general population. It is possible that those admitted with respiratory disease were more frail than those without, creating the impression that respiratory disease confers greater risk than is actually the case.^19^ To our knowledge, ISARIC is the only study to have examined risks in people with interstitial lung disease.^5^ In patients admitted to hospital, people with interstitial lung disease matched for age, sex, and comorbidity had an HR of 1·60 (95% CI 1·17–2·18) for death. The risks were slightly higher in people with idiopathic pulmonary fibrosis (1·74 [1·16–2·60]) than in those with other forms of interstitial lung disease (1·50 [1·02–2·21]).^5^ These estimates are slightly higher than the estimates we produced, but we adjusted for more potentially confounding variables and included all people with interstitial lung disease in the population. The only representative population cohort we know to have reported on these associations is the OpenSAFELY study^10^ of 17 million patients in England. It reported on risk of death for people with asthma who had not used oral steroids within the past 4 months (HR 0·99 [0·93–1·05]), those who had used oral steroids within the past 4 months (1·13 [1·01–1·26]), and all other respiratory diseases (1·63 [1·55–1·71]). Although we did not differentiate people with asthma by oral steroid use, the estimates for asthma are remarkably similar to those in our cohort. A US cohort matched people with asthma infected with SARS-CoV-2 to people without asthma with a SARS-CoV-2 infection.^20^ They reported that the risk of hospitalisation was similar in the two groups, ICU admission non-significantly less likely in patients without asthma who had a SARS-CoV-2 infection, and death markedly less likely in people with asthma and COVID-19. Our findings on airways disease have expanded the current literature considerably, showing that asthma is associated with a very small increase in risk. The absence of a substantial association might seem surprising, given that respiratory viral infection is the main cause of exacerbations of asthma.^21^ However, the finding is compatible with data from a small cohort of patients with asthma who were hospitalised with COVID-19, which showed no evidence that COVID-19 exacerbated asthma.^21^, ^22^, ^23^

No previous study has reported that the risk associated with asthma appears to be higher in women than in men. These results might reflect the fact that women have a higher prevalence of asthma and are at increased risk of severe asthma,^22^, ^23^ whereas men (whether they have asthma or not) are at higher risk of severe COVID-19.^24^ It could be that women's tendency towards severe asthma might explain why asthma appears to confer a slightly higher risk of severe COVID-19 for women than men, but any increase in risk for severe COVID-19 in women with asthma is low.

There is also a small amount of evidence on the association between ICS use and COVID-19. A systematic review reported that, in people admitted to hospital with severe COVID-19, use of ICS was associated with recovery, shorter duration of hospital stay, but no evidence of association with need for ICU care or death.^25^ Since then, two studies in community cohorts have been reported. A Spanish cohort found that people using ICS were at lower risk of being hospitalised with COVID-19 than people who did not use ICS, OR 0·58 (95% CI 0·44–0·77).^26^ The OpenSAFELY cohort^10^ reported that in people with COPD, ICS use had a higher HR for death from COVID-19 of 1·39 (95% CI 1·10–1·76) than did long-acting β-agonists and long-acting muscarinic antagonist use.^10^ In people with asthma, ICS use had an HR of 1·14 (0·85–1·54) for low or medium dose, and 1·55 (1·10–2·18) for high-dose ICS use, compared with short-acting β-agonist only. We used a different approach for the analyses, but the OpenSAFELY^10^ estimates are similar to the ones we report for COVID-19 death associated with steroid use, adjusted for other respiratory illnesses, medication use, and other potential confounders. However, we also report estimates for the associations with hospitalisation and ICU care, which suggest similar relative hazards to those for death from COVID-19. Because ICS use will typically be higher in people with more severe disease, these data from community cohorts taken together suggest that ICS use does not influence the likelihood of severe COVID-19.

From the beginning of the pandemic, governments have warned people with respiratory disease that they are at higher risk of severe disease, and these warnings have been reflected by charities for people with respiratory disease.^27^, ^28^ Our results do suggest that such diseases confer increased risk, but that the extent of this increase in risk, particularly for people with asthma, might be less than originally thought. COPD and interstitial lung disease appear to confer a 30–50% increased risk of severe COVID-19, while asthma confers a 10–30% increase compared with those without disease. By contrast, in the same models, being male was associated with a 46% increased risk of hospitalisation, a three-times higher risk of ICU admission, and an 86% increased risk of death compared with being female. People with respiratory disease feel particularly anxious about their disease predisposing them to severe COVID-19.^29^ One study found that anxiety and depression increased during lockdown to a significantly greater extent in people with asthma than in those without.^30^ We know of no evidence that being a man generates the same degree of concern about risk of COVID-19 as that reported by people with asthma, when it presents an objectively higher risk. Our use of normal risks of mortality also put these risks in context. The risk of death from COVID-19 perhaps caused by underlying respiratory disease was far lower than the ordinary risk of death in the 4 months (January to April, 2020) that included the peak of the SARS-CoV-2 epidemic in England. Although this could be partly due to behavioural responses of people with respiratory disease, the noticeably higher risk in people with diabetes,^31^ also subject to the same warnings regarding their susceptibility to severe COVID-19, together with our analyses suggest that this is not the whole explanation.

The assumption that pre-existing respiratory disease would inevitably lead to an increased risk from COVID-19 was reasonable at the start of the pandemic, but as data continue to emerge, these assumptions might need to be revisited, and advice to people with respiratory disease from governments and charities should perhaps be substantially more reassuring.

For the **QResearch database** see www.qresearch.org

## Data sharing

Information on how to access the QResearch database can be found online. The protocol is published and the statistical analysis plan is available on reasonable request to the corresponding author.

## References

[1] Halpin DMG, Faner R, Sibila O, Badia JR, Agusti A (2020). Do chronic respiratory diseases or their treatment affect the risk of SARS-CoV-2 infection?. Lancet Respir Med.

[2] Hartmann-Boyce J, Gunnell J, Drake J (2020). Asthma and COVID-19: review of evidence on risks and management considerations. BMJ Evid Based Med.

[3] Sanchez-Ramirez DC, Mackey D (2020). Underlying respiratory diseases, specifically COPD, and smoking are associated with severe COVID-19 outcomes: a systematic review and meta-analysis. Respir Med.

[4] Griffith GJ, Hermani G, Herbert A (2020). We should be cautious about associations of patient characteristics with COVID-19 outcomes that are identified in hospitalised patients. https://www.hdruk.ac.uk/news/we-should-be-cautious-about-associations-of-patient-characteristics-with-covid-19-outcomes-that-are-identified-in-hospitalised-patients/.

[5] Drake TM, Docherty AB, Harrison EM (2020). Outcome of hospitalization for COVID-19 in patients with interstitial lung disease: an international multicenter study. Am J Respir Crit Care Med.

[6] Jeon S, Ko M, Lee J (2020). Identification of antiviral drug candidates against SARS-CoV-2 from FDA-approved drugs. Antimicrob Agents Chemother.

[7] Yamaya M, Nishimura H, Deng X (2020). Inhibitory effects of glycopyrronium, formoterol, and budesonide on coronavirus HCoV-229E replication and cytokine production by primary cultures of human nasal and tracheal epithelial cells. Respir Investig.

[8] Halpin DMG, Singh D, Hadfield RM (2020). Inhaled corticosteroids and COVID-19: a systematic review and clinical perspective. Eur Respir J.

[9] Yang M, Zhang Y, Chen H, Lin J, Zeng J, Xu Z (2019). Inhaled corticosteroids and risk of upper respiratory tract infection in patients with asthma: a meta-analysis. Infection.

[10] Schultze A, Walker AJ, MacKenna B (2020). Risk of COVID-19-related death among patients with chronic obstructive pulmonary disease or asthma prescribed inhaled corticosteroids: an observational cohort study using the OpenSAFELY platform. Lancet Respir Med.

[11] Aveyard P, Lindson N, Gao M (2020). Associations between COVID-19 infection, tobacco smoking and nicotine use, common respiratory conditions and inhaled corticosteroids: a prospective QResearch-Case Mix Programme data linkage study January-May 2020. medRxiv.

[12] Demedts M, Wells AU, Antó JM (2001). Interstitial lung diseases: an epidemiological overview. Eur Respir J Suppl.

[13] Hewitt J, Carter B, Vilches-Moraga A (2020). The effect of frailty on survival in patients with COVID-19 (COPE): a multicentre, European, observational cohort study. Lancet Public Health.

[14] Spiegelhalter D (2020). Use of “normal” risk to improve understanding of dangers of covid-19. BMJ.

[15] Office for National Statistics (2020). National life tables, UK: 2016 to 2018. https://www.ons.gov.uk/peoplepopulationandcommunity/birthsdeathsandmarriages/lifeexpectancies/bulletins/nationallifetablesunitedkingdom/2016to2018.

[16] Cole SR, Platt RW, Schisterman EF (2009). Illustrating bias due to conditioning on a collider. Int J Epidemiol.

[17] Greenhalgh T, Knight M, A'Court C, Buxton M, Husain L (2020). Management of post-acute covid-19 in primary care. BMJ.

[18] Sudre CH, Murray B, Varsavsky T (2020). Attributes and predictors of Long-COVID: analysis of COVID cases and their symptoms collected by the COVID symptoms study app. medRxiv.

[19] Sterne JAC (2020). We should be cautious about associations of patient characteristics with COVID-19 outcomes that are identified in hospitalised patients. Health Data Research UK. https://www.hdruk.ac.uk/news/we-should-be-cautious-about-associations-of-patient-characteristics-with-covid-19-outcomes-that-are-identified-in-hospitalised-patients/.

[20] Robinson LB, Wang L, Fu X (2021). COVID-19 severity in asthma patients: a multi-center matched cohort study. J Asthma.

[21] Oliver BGG, Robinson P, Peters M, Black J (2014). Viral infections and asthma: an inflammatory interface?. Eur Respir J.

[22] Zein JG, Erzurum SC (2015). Asthma is different in women. Curr Allergy Asthma Rep.

[23] Leynaert B, Sunyer J, Garcia-Esteban R (2012). Gender differences in prevalence, diagnosis and incidence of allergic and non-allergic asthma: a population-based cohort. Thorax.

[24] Park R, Chidharla A, Mehta K, Sun W, Wulff-Burchfield E, Kasi A (2020). Sex-bias in COVID-19-associated illness severity and mortality in cancer patients: a systematic review and meta-analysis. EClinicalMedicine.

[25] Cheng W, Li Y, Cui L (2020). Efficacy and safety of corticosteroid treatment in patients with COVID-19: a systematic review and meta-analysis. Front Pharmacol.

[26] Izquierdo JL, Almonacid C, González Y (2020). The impact of COVID-19 on patients with asthma. Eur Respir J.

[27] NHS England (2020). Who's at higher risk from coronavirus. https://www.nhs.uk/conditions/coronavirus-covid-19/people-at-higher-risk/whos-at-higher-risk-from-coronavirus/.

[28] US CDC (2020). Coronavirus disease 2019 (COVID-19): people with certain medical conditions. https://www.cdc.gov/coronavirus/2019-ncov/need-extra-precautions/people-with-medical-conditions.html.

[29] Philip KEJ, Lonergan B, Cumella A, Farrington-Douglas J, Laffan M, Hopkinson NS (2020). COVID-19 related concerns of people with long-term respiratory conditions: a qualitative study. BMC Pulm Med.

[30] Higbee DH, Nava G, Kwong ASF, Dodd JW, Granell R (2020). The impact of asthma on mental health and wellbeing during COVID-19 lockdown. medRxiv.

[31] Barron E, Bakhai C, Kar P (2020). Associations of type 1 and type 2 diabetes with COVID-19-related mortality in England: a whole-population study. Lancet Diabetes Endocrinol.

