# Supplementary material for: Association between pre-existing respiratory disease and its treatment, and severe COVID-19: a population cohort study
Source: Lancet Respir Med. 2021 Aug;9(8):909–23. doi: 10.1016/S2213-2600(21)00095-3 (PMC8016404; doi:10.1016/S2213-2600(21)00095-3)
Supplement: Supplementary appendix [file mmc1.pdf]

THE LANCET
Respiratory Medicine

Supplementary appendix

This appendix formed part of the original submission and has been peer reviewed. We post it as supplied by the authors.

Supplement to: Aveyard P, Gao M, Lindson N, et al. Association between pre-existing respiratory disease and its treatment, and severe COVID-19: a population cohort study. *Lancet Respir Med* 2021; published online April 1. http://dx.doi.org/10.1016/S2213-2600(21)00095-3.

Appendix Figure 1 Directed acyclic graphs of the associations between respiratory disease and severe COVID-19

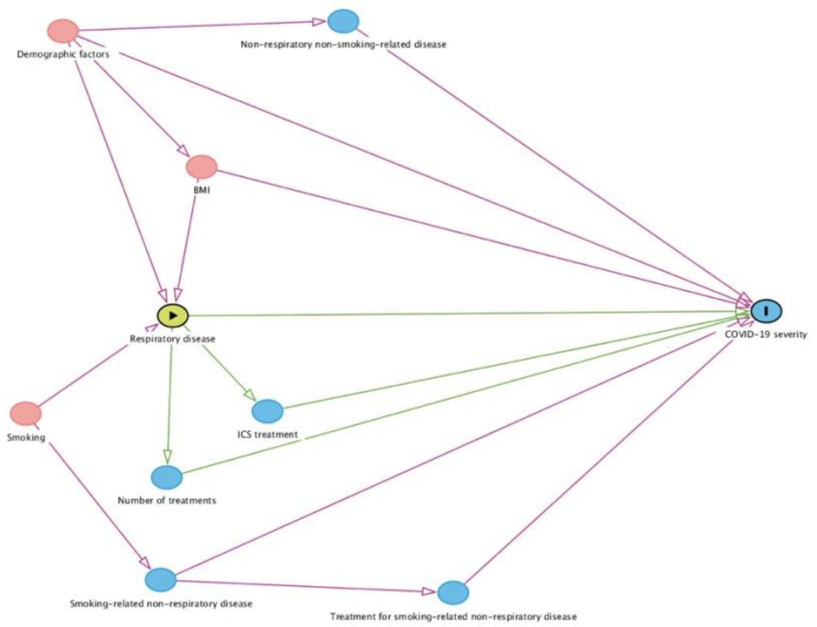

Appendix Figure 2 Directed acyclic graph for the association between ICS use and severe COVID-19

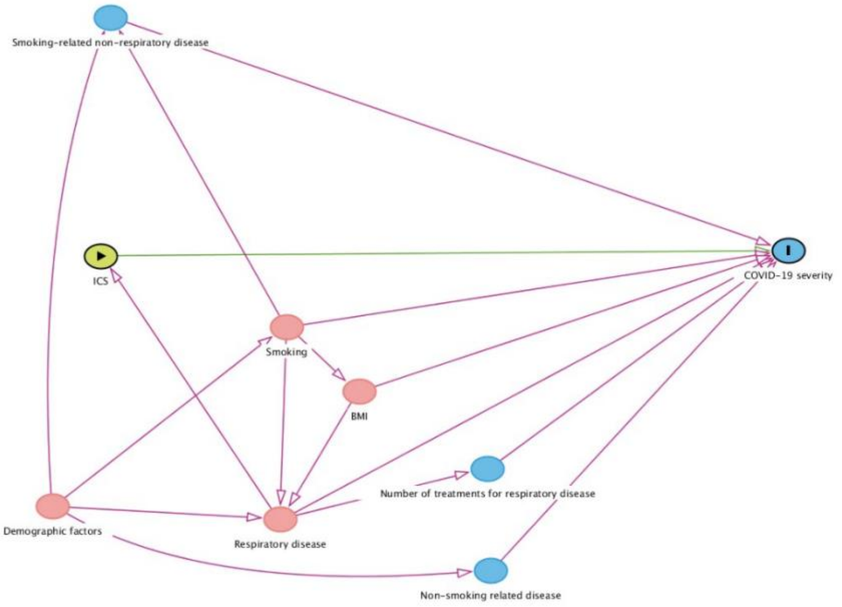

Table 1 Appendix Characteristics of the population

Factor	Whole population (n=8,256,161)	Number admitted to hospital with COVID-19 (n=14,479)	Number admitted to ICU with COVID-19 (n=1,542)	Number of Covid-19 deaths (n=5,956)
Respiratory disease				
COPD	193520 (2.3%)	1555 (10.7%)	59 (3.8%)	811 (13.6%)
Asthma	1090028 (13.2%)	2266 (15.7%)	213 (13.8%)	762 (12.8%)
Active asthma	535126 (6.5%)	1720 (11.9%)	165 (10.7%)	602 (10.1%)
Severe asthma	385702 (4.7%)	1369 (9.5%)	124 (8.0%)	476 (8.0%)
Bronchiectasis	41271 (0.5%)	319 (2.2%)	18 (1.2%)	138 (2.3%)
Cystic fibrosis	2081 (<1%)	5 (<1%)	0 (0.0%)	0 (0.0%)
Sarcoidosis	17624 (0.2%)	84 (0.6%)	10 (0.6%)	32 (0.5%)
Extrinsic allergic alveolitis	2331 (<1%)	16 (0.1%)	*	8 (0.1%)
Idiopathic pulmonary fibrosis	7454 (0.1%)	110 (0.8%)	6 (0.4%)	62 (1.0%)
Other interstitial lung disease	5677 (0.1%)	73 (0.5%)	*	45 (0.8%)
Lung cancer	10792 (0.1%)	139 (1.0%)	*	60 (1.0%)
Any respiratory disease	1271108 (15.4%)	3696 (25.5%)	275 (17.8%)	1485 (24.9%)
Airways medication				
Regular inhaled corticosteroid use	450784 (5.5%)	1960 (13.5%)	166 (10.8%)	784 (13.2%)
Leukotriene receptor antagonists	41728 (0.5%)	228 (1.6%)	27 (1.8%)	69 (1.2%)
Short-acting beta agonist	495909 (6.0%)	2357 (16.3%)	163 (10.6%)	988 (16.6%)
Anticholinergic	93041 (1.1%)	820 (5.7%)	36 (2.3%)	400 (6.7%)
Theophylline/xanthine	8533 (0.1%)	114 (0.8%)	*	39 (0.7%)
Combined beta agonist and inhaled corticosteroid	21976 (0.3%)	156 (1.1%)	*	89 (1.5%)
Combined long-acting beta agonist and inhaled corticosteroid	284933 (3.5%)	1499 (10.4%)	119 (7.7%)	591 (9.9%)
Long-acting beta agonist	8793 (0.1%)	62 (0.4%)	*	26 (0.4%)
Combined long-acting anti-muscarinic and long-acting beta agonist	25204 (0.3%)	167 (1.2%)	5 (0.3%)	92 (1.5%)
Inhaled corticosteroids alone	180076 (2.2%)	565 (3.9%)	59 (3.8%)	239 (4.0%)
Using 3 or more airways medications	382265(4.6%)	1918(13.3%)	139(9.0%)	796(13.4%)
Smoking status				
Never smoked	4718435 (57.2%)	8133 (56.2%)	937 (60.8%)	3108 (52.2%)
Formerly smoked	1748966 (21.2%)	5006 (34.6%)	518 (33.6%)	2342 (39.3%)
Smokes 1-9 cigarettes/day	1097213 (13.3%)	902 (6.2%)	60 (3.9%)	295 (5.0%)

Smokes 10-19 cigarettes/day	208767 (2.5%)	120 (0.8%)	5 (0.3%)	40 (0.7%)
Smokes ≥20 cigarettes/day	96153 (1.2%)	92 (0.6%)	7 (0.5%)	32 (0.5%)
Unknown smoking status	386627 (4.7%)	226 (1.6%)	15 (1.0%)	139 (2.3%)
Age				
Mean age (SD)	48.2 (18.6)	69.9 (17.7)	59.5 (12.5)	80.2 (12.0)
Age group				
20-39	3171742 (38.4%)	1029 (7.1%)	107 (6.9%)	40 (0.7%)
40-59	2752758 (33.3%)	2938 (20.3%)	621 (40.3%)	366 (6.1%)
60-79	1823544 (22.1%)	5139 (35.5%)	766 (49.7%)	1887 (31.7%)
80-100	508117 (6.2%)	5373 (37.1%)	48 (3.1%)	3663 (61.5%)
Sex- men (%)	4111200 (49.8%)	8038 (55.5%)	1132 (73.4%)	3423 (57.5%)
Ethnicity as recorded				
White	5359536 (64.9%)	9193 (63.5%)	755 (49.0%)	4019 (67.5%)
Indian	227767 (2.8%)	534 (3.7%)	81 (5.3%)	164 (2.8%)
Pakistani	148399 (1.8%)	323 (2.2%)	52 (3.4%)	93 (1.6%)
Bangladeshi	111077 (1.3%)	223 (1.5%)	47 (3.0%)	91 (1.5%)
Other Asian	145010 (1.8%)	319 (2.2%)	83 (5.4%)	76 (1.3%)
Caribbean	93339 (1.1%)	529 (3.7%)	63 (4.1%)	217 (3.6%)
Black African	198427 (2.4%)	594 (4.1%)	129 (8.4%)	156 (2.6%)
Chinese	82598 (1.0%)	64 (0.4%)	14 (0.9%)	25 (0.4%)
Other	306060 (3.7%)	568 (3.9%)	113 (7.3%)	137 (2.3%)
Not recorded	1583948 (19.2%)	2132 (14.7%)	205 (13.3%)	978 (16.4%)
Ethnicity as analysed				
White	5359536 (64.9%)	9193 (63.5%)	755 (49.0%)	4019 (67.5%)
Asian	632253 (7.7%)	1399 (9.7%)	263 (17.1%)	424 (7.1%)
Black	291766 (3.5%)	1123 (7.8%)	192 (12.5%)	373 (6.3%)
Chinese	82598 (1.0%)	64 (0.4%)	14 (0.9%)	25 (0.4%)
Others/not recorded	1890008 (22.9%)	2700 (18.6%)	318 (20.6%)	1115 (18.7%)
Townsend deprivation score				
1 (most affluent)	1881317 (22.9%)	2777 (19.2%)	246 (16.0%)	1235 (20.8%)
2	1821068 (22.2%)	2850 (19.7%)	265 (17.2%)	1170 (19.7%)
3	1629714 (19.8%)	2957 (20.5%)	306 (19.9%)	1335 (22.5%)
4	1483531 (18.1%)	2824 (19.6%)	304 (19.8%)	1084 (18.2%)
5 (most deprived)	1402402 (17.1%)	3035 (21.0%)	417 (27.1%)	1121 (18.9%)
Not recorded	38129 (0.5%)	36 (0.2%)	*	11 (0.2%)

Region				
East Midlands	220879 (2.7%)	216 (1.5%)	15 (1.0%)	93 (1.6%)
East of England	296231 (3.6%)	490 (3.4%)	45 (2.9%)	209 (3.5%)
London	2059744 (24.9%)	4963 (34.3%)	693 (44.9%)	1770 (29.7%)
North East	195156 (2.4%)	321 (2.2%)	28 (1.8%)	109 (1.8%)
North West	1470043 (17.8%)	2773 (19.2%)	258 (16.7%)	1268 (21.3%)
South Central	1104630 (13.4%)	1720 (11.9%)	148 (9.6%)	800 (13.4%)
South East	925796 (11.2%)	1298 (9.0%)	141 (9.1%)	577 (9.7%)
South West	900553 (10.9%)	797 (5.5%)	68 (4.4%)	299 (5.0%)
West Midlands	777225 (9.4%)	1514 (10.5%)	111 (7.2%)	654 (11.0%)
Yorkshire & Humber	305904 (3.7%)	387 (2.7%)	35 (2.3%)	177 (3.0%)
Hypertension	1407799 (17.1%)	2633 (18.2%)	65 (4.2%)	2222 (37.3%)
Coronary heart disease	292839 (3.5%)	2414 (16.7%)	128 (8.3%)	1373 (23.1%)
Stroke	177763 (2.2%)	1822 (12.6%)	51 (3.3%)	1144 (19.2%)
Atrial fibrillation	201250 (2.4%)	1750 (12.1%)	50 (3.2%)	1101 (18.5%)
Type 2 diabetes	555416 (6.7%)	1993 (13.8%)	50 (3.2%)	1100 (18.5%)
Type 1 diabetes	45947 (0.6%)	3537 (24.4%)	174 (11.3%)	1962 (32.9%)
Chronic kidney disease	336880 (4.1%)	4237 (29.3%)	480 (31.1%)	1935 (32.5%)
Chronic liver disease	47141 (0.6%)	272 (1.9%)	30 (1.9%)	85 (1.4%)
Chronic neurological disease	256538 (3.1%)	286 (2.0%)	16 (1.0%)	98 (1.6%)
BMI groups				
BMI<18.5	220706 (2.7%)	361 (2.5%)	5 (0.3%)	274 (4.6%)
BMI 18.5-24.9	2742969 (33.2%)	3660 (25.3%)	198 (12.8%)	1869 (31.4%)
BMI 25-29.9	2315214 (28.0%)	4602 (31.8%)	491 (31.8%)	1754 (29.4%)
BMI 30-34.9	1076005 (13.0%)	2823 (19.5%)	407 (26.4%)	965 (16.2%)
BMI 35-39.9	406529 (4.9%)	1276 (8.8%)	222 (14.4%)	384 (6.4%)
BMI ≥40	203717 (2.5%)	793 (5.5%)	135 (8.8%)	234 (3.9%)
BMI not recorded	1291021 (15.6%)	964 (6.7%)	84 (5.4%)	476 (8.0%)

* Data not presented as fewer than 5 people had this outcome

Table 2 Appendix Characteristics of the population by ICS use, asthma and lung disease combined

Factor	People using ICS (n= 450784)	People with asthma(n=1090028)	People with COPD(n=193520)	People with all other respiratory disease (n=30710)
Airways medication				
Leukotriene receptor antagonists	35232 (7.8%)	37716 (3.5%)	6696 (3.5%)	497 (1.6%)
Short-acting beta agonist	325965 (72.3%)	345304 (31.7%)	127408 (65.8%)	5534 (18.0%)
Anticholinergic	68472 (15.2%)	38014 (3.5%)	75310 (38.9%)	1955 (6.4%)
Theophylline/xanthine	6550 (1.5%)	6029 (0.6%)	5557 (2.9%)	150 (0.5%)
Combined beta agonist and inhaled corticosteroid	2904 (0.6%)	3295 (0.3%)	20055 (10.4%)	528 (1.7%)
Combined long-acting beta agonist and inhaled corticosteroid	284933 (63.2%)	229517 (21.1%)	78322 (40.5%)	3852 (12.5%)
Long-acting beta agonist	5452 (1.2%)	5123 (0.5%)	4064 (2.1%)	118 (0.4%)
Combined long-acting anti-muscarinic and long-acting beta agonist	6193 (1.4%)	3605 (0.3%)	23194 (12.0%)	584 (1.9%)
Inhaled corticosteroids alone	180076 (39.9%)	149465 (13.7%)	17522 (9.1%)	1563 (5.1%)
Using 3 or more airways medications	342982(75.6%)	288196(26.5%)	113446(58.7%)	4558(14.9%)
Smoking status				
Never smoked	222224 (49.3%)	624797 (57.3%)	23935 (12.4%)	17406 (56.7%)
Formerly smoked	147629 (32.7%)	257566 (23.6%)	104638 (54.1%)	10497 (34.2%)
Smokes 1-9 cigarettes/day	62939 (14.0%)	153208 (14.1%)	49545 (25.6%)	2123 (6.9%)
Smokes 10-19 cigarettes/day	10585 (2.3%)	27515 (2.5%)	8494 (4.4%)	347 (1.1%)
Smokes ≥20 cigarettes/day	6631 (1.5%)	12650 (1.2%)	6736 (3.5%)	207 (0.7%)
Unknown smoking status	776 (0.2%)	14292 (1.3%)	172 (0.1%)	130 (0.4%)
Age				
Mean age (SD)	57.55 (18.03)	46.65 (18.34)	70.96 (11.44)	66.11 (14.96)
Age group				
20-39	84077 (18.7%)	459751 (42.2%)	1105 (0.6%)	1543 (5.0%)
40-59	148307 (32.9%)	352853 (32.4%)	31175 (16.1%)	8607 (28.0%)
60-79	167010 (37.0%)	218881 (20.1%)	115046 (59.4%)	14175 (46.2%)
80-100	51390 (11.4%)	58543 (5.4%)	46194 (23.9%)	6385 (20.8%)
Sex- men (%)	190411 (42.2%)	518531 (47.6%)	100844 (52.1%)	16203 (52.8%)
Ethnicity as recorded				
White	343711 (76.2%)	784083 (71.9%)	161376 (83.4%)	21268 (69.3%)
Indian	9346 (2.1%)	23143 (2.1%)	1428 (0.7%)	993 (3.2%)

Pakistani	8993 (2.0%)	19427 (1.8%)	1236 (0.6%)	573 (1.9%)
Bangladeshi	5462 (1.2%)	12409 (1.1%)	1015 (0.5%)	300 (1.0%)
Other Asian	5320 (1.2%)	13035 (1.2%)	784 (0.4%)	394 (1.3%)
Caribbean	5344 (1.2%)	14158 (1.3%)	1309 (0.7%)	840 (2.7%)
Black African	4863 (1.1%)	14194 (1.3%)	591 (0.3%)	729 (2.4%)
Chinese	963 (0.2%)	3503 (0.3%)	178 (0.1%)	41 (0.1%)
Other	9830 (2.2%)	31443 (2.9%)	1743 (0.9%)	946 (3.1%)
Not recorded	56952 (12.6%)	174633 (16.0%)	23860 (12.3%)	4626 (15.1%)
Ethnicity as analysed				
White	343711 (76.2%)	784083 (71.9%)	161376 (83.4%)	21268 (69.3%)
Asian	29121 (6.5%)	68014 (6.2%)	4463 (2.3%)	2260 (7.4%)
Black	10207 (2.3%)	28352 (2.6%)	1900 (1.0%)	1569 (5.1%)
Chinese	963 (0.2%)	3503 (0.3%)	178 (0.1%)	41 (0.1%)
Others/not recorded	66782 (14.8%)	206076 (18.9%)	25603 (13.2%)	5572 (18.1%)
Townsend deprivation score				
1 (most affluent)	108162 (24.0%)	259097 (23.8%)	36437 (18.8%)	8735 (28.4%)
2	105183 (23.3%)	253449 (23.3%)	41488 (21.4%)	7562 (24.6%)
3	93628 (20.8%)	225539 (20.7%)	43088 (22.3%)	5961 (19.4%)
4	81210 (18.0%)	192184 (17.6%)	40193 (20.8%)	4743 (15.4%)
5 (most deprived)	61014 (13.5%)	154828 (14.2%)	31946 (16.5%)	3638 (11.8%)
Not recorded	1587 (0.4%)	4931 (0.5%)	368 (0.2%)	71 (0.2%)
Region				
East Midlands	9741 (2.2%)	27906 (2.6%)	3780 (2.0%)	601 (2.0%)
East of England	17295 (3.8%)	42244 (3.9%)	6781 (3.5%)	1286 (4.2%)
London	79010 (17.5%)	214852 (19.7%)	30647 (15.8%)	6317 (20.6%)
North East	11885 (2.6%)	27984 (2.6%)	7104 (3.7%)	624 (2.0%)
North West	102435 (22.7%)	219030 (20.1%)	49069 (25.4%)	5955 (19.4%)
South Central	60539 (13.4%)	156435 (14.4%)	22460 (11.6%)	4383 (14.3%)
South East	54050 (12.0%)	118760 (10.9%)	23270 (12.0%)	3357 (10.9%)
South West	50334 (11.2%)	134231 (12.3%)	22912 (11.8%)	3788 (12.3%)
West Midlands	48730 (10.8%)	107417 (9.9%)	19561 (10.1%)	3320 (10.8%)
Yorkshire & Humber	16765 (3.7%)	41169 (3.8%)	7936 (4.1%)	1079 (3.5%)
Hypertension	143355 (31.8%)	191752 (17.6%)	90448 (46.7%)	12642 (41.2%)
Coronary heart disease	35671 (7.9%)	41125 (3.8%)	34506 (17.8%)	4085 (13.3%)
Stroke	19790 (4.4%)	24165 (2.2%)	18163 (9.4%)	2178 (7.1%)

Atrial fibrillation	22760 (5.0%)	26814 (2.5%)	20832 (10.8%)	2733 (8.9%)
Type 2 diabetes	59047 (13.1%)	82278 (7.5%)	37718 (19.5%)	5907 (19.2%)
Type 1 diabetes	37308 (8.3%)	45556 (4.2%)	31029 (16.0%)	4988 (16.2%)
Chronic kidney disease	3309 (0.7%)	6907 (0.6%)	1327 (0.7%)	338 (1.1%)
Chronic liver disease	5265 (1.2%)	7422 (0.7%)	4335 (2.2%)	556 (1.8%)
Chronic neurological disease	21881 (4.9%)	38884 (3.6%)	14675 (7.6%)	1948 (6.3%)
BMI groups				
BMI<18.5	9553 (2.1%)	32821 (3.0%)	9062 (4.7%)	537 (1.7%)
BMI 18.5-24.9	125172 (27.8%)	350914 (32.2%)	61518 (31.8%)	8299 (27.0%)
BMI 25-29.9	145554 (32.3%)	315370 (28.9%)	61169 (31.6%)	10977 (35.7%)
BMI 30-34.9	88949 (19.7%)	169695 (15.6%)	35865 (18.5%)	6022 (19.6%)
BMI 35-39.9	41383 (9.2%)	76970 (7.1%)	14746 (7.6%)	2356 (7.7%)
BMI ≥40	26849 (6.0%)	47464 (4.4%)	7667 (4.0%)	1178 (3.8%)
BMI not recorded	13324 (3.0%)	96794 (8.9%)	3493 (1.8%)	1341 (4.4%)

* Data not presented as fewer than 5 people had this outcome
